# PanRV: Pangenome-reverse vaccinology approach for identifications of potential vaccine candidates in microbial pangenome

**DOI:** 10.1186/s12859-019-2713-9

**Published:** 2019-03-12

**Authors:** Kanwal Naz, Anam Naz, Shifa Tariq Ashraf, Muhammad Rizwan, Jamil Ahmad, Jan Baumbach, Amjad Ali

**Affiliations:** 1 Atta-ur-Rahman School of Applied Biosciences (ASAB), National University of Sciences and Technology (NUST), H-12, Islamabad, 44000 Pakistan; 2 Research Center for Modeling and Simulation (RCMS), National University of Sciences and Technology (NUST), H-12, Islamabad, Pakistan; 3 Chair of Experimental Bioinformatics, TUM School of Life Sciences Weihenstephan, Technical University of Munich, Munchen, Germany; 4 Department of Computer Science and Information Technology, University of Malakand, Chakdara, Khyber Pakhtunkhwa Pakistan

**Keywords:** PanRV, Pangenome, Core genome, Reverse vaccinology, Microbial species, Vaccine targets, And therapeutic targets

## Abstract

**Background:**

A revolutionary diversion from classical vaccinology to reverse vaccinology approach has been observed in the last decade. The ever-increasing genomic and proteomic data has greatly facilitated the vaccine designing and development process. Reverse vaccinology is considered as a cost-effective and proficient approach to screen the entire pathogen genome. To look for broad-spectrum immunogenic targets and analysis of closely-related bacterial species, the assimilation of pangenome concept into reverse vaccinology approach is essential. The categories of species pangenome such as core, accessory, and unique genes sets can be analyzed for the identification of vaccine candidates through reverse vaccinology.

**Results:**

We have designed an integrative computational pipeline term as “PanRV” that employs both the pangenome and reverse vaccinology approaches. PanRV comprises of four functional modules including i) Pangenome Estimation Module (PGM) ii) Reverse Vaccinology Module (RVM) iii) Functional Annotation Module (FAM) and iv) Antibiotic Resistance Association Module (ARM). The pipeline is tested by using genomic data from 301 genomes of *Staphylococcus aureus* and the results are verified by experimentally known antigenic data.

**Conclusion:**

The proposed pipeline has proved to be the first comprehensive automated pipeline that can precisely identify putative vaccine candidates exploiting the microbial pangenome. PanRV is a Linux based package developed in JAVA language. An executable installer is provided for ease of installation along with a user manual at https://sourceforge.net/projects/panrv2/.

**Electronic supplementary material:**

The online version of this article (10.1186/s12859-019-2713-9) contains supplementary material, which is available to authorized users.

## Background

Microbial species are rapidly evolving and acquiring multi-drug resistance, making existing therapies ineffective [1]. Hence, there is a need to identify broad-spectrum therapeutic targets, which will be effective against a range of closely related microbial pathogens. Advancements in genome sequencing technologies and high-throughput bioinformatics analyses have assisted the basic in-vivo vaccine design via *in-silico* practices [2]. The genomes of thousands of pathogenic microbes have been sequenced so far, and are available for scientific exploration such as antibiotic resistance determination and finding alternative therapeutic targets [3]. Due to genomic diversity in bacterial species, a large number of variable genes accumulate in species gene pool ultimately resulting in the species pangenome expansion [4]. Therefore, considering a single representative (genome) from such a species is not sufficient to estimate the exact pangenome and is unfavorable to be targeted for broad-spectrum therapeutics. On the other hand, closely related bacterial species share a large number of genomic contents and hence remain less diverge. Thus, pangenome analysis is a suitable approach for estimating the diversity in strains of the same species and in rarely in Genera [4]. The bacterial pangenome concept was introduced in 2005 for analyzing pathogenic bacterial species and can be defined as the entire set of genes in a group of representative strains of the same genus/species. Pangenome can be classified into conserved core genome (genes/proteins present in all the genomes), a dispensable genome (set of genes shared by multiple genomes, but not all) and unique genes (genes confined to individual organism/genomes) [5]. This approach is considered the best to explore and analyze multiple pathogenic bacterial species or strains (genomes) and to estimate the conserved core, dispensable and unique gene families [6]**.** Also give clues of the nature of the pangenome of a species, whether it is still open or closed.

Almost at the same time, reverse vaccinology (RV) emerges as one of the applied approaches to assess the genomic sequences for prediction of novel candidate proteins and their immunogenic epitopes which may elicit protective immune responses [7, 8]. The RV is a stepwise computational screening process that analyzes each protein from the whole set of bacterial proteome for its antigenic and immunogenic potentials. A significant decrease in time and cost is observed using this strategy instead of culturing the whole microorganism to identify potential vaccine candidates (PVCs) [9]. RV approach has been applied successfully to analyze several pathogenic species and a number of PVCs are predicted, these PVCs are then tested in-vivo which led to the development of licensed protein vaccine. The first milestone of RV is a vaccine development against *Neisseria meningitidis* serogroup B (MenB) pathogen [10], where five antigenic protein components including GNA1030, GNA1870, GNA2091 GNA2132 and NadA, were identified. This implication of RV approach was particularly acknowledged in case of MenB, as the vaccine developed earlier using capsular polysaccharides was found ineffective due to cross-reactivity against human tissues [11]. Subsequently, a progressive success has been observed in case of pathogens including *Helicobacter pylori* [12], *Streptococcus pneumoniae* [13], *Porphyromonas gingivalis* [14], *Chlamydia pneumoniae* [15] and *Bacillus anthracis* [16].

In the context of computational tools, there are various online tools available which have implemented the RV approach, these tools include Vaxign [17], VaxiJen [18], and Jenner-predict [19]. Since they are web-based tools, therefore, have limitations of analysis time or the input data size. There are tools available in packages such as VacSol [20], NERVE [21] and Vacceed [22], however, they also have few limitations. For example, NERVE only focuses on adhesive proteins where many significant secreted proteins are overlooked which can be good vaccine targets [20]. Additionally, NERVE and Jenner-predict are having functional issues which may be due to a lack of proper maintenance. While Vacceed provides limited information about the nature of predicted targets such as pathogenicity and functional annotation [20]. Furthermore, these existing tools have the limitation to analyze a single genome (strain) at a time, hence the prediction of a broad-spectrum therapeutic target(s) remained a challenge [6].

In order to expedite the in-vivo vaccine development process and to design universal vaccines, we aimed to devise a faster, efficient and cost-effective *in-silico* framework by combining the notions of pangenome (Pan) and reverse vaccinology (RV) into a single comprehensive pipeline termed as PanRV. The pipeline employed pangenome concept into an RV approach so that genomic repertoire of all the available isolates of a species can be exploited to identify vaccine targets. Therefore, it is a significant step towards the prioritization of broad-spectrum drugs and vaccine candidates. The pipeline is tested on selected bacterial species and equally applicable to all bacterial species. The pipeline integrated a number of standalone bioinformatics tools and databases, the list of tools and databases is provided in Table 1. The PanRV is designed to have multiple functional modules and provide an interactive Graphical User Interface (GUI). The two major modules include 1) Pangenome Estimation Module (PGM) and 2) Reverse Vaccinology Module (RVM). Other modules include the 3) Functional Annotation Module (FAM) and 4) Antibiotic Resistance Association Module (ARM). After estimation of the pangenome through PGM, users may analyze selected category such as the pan, core, dispensable or unique genes for further screening of potential therapeutic targets (vaccine candidates) using RVM. Further, functional annotation and resistance analysis of the candidates can also be performed by FAM and ARM modules. A detailed comparison of different tools with PanRV based on their specific features (functionalities) is also provided in Table 2.

**Table 1:** Tools and databases implemented and integrated into the PanRV modules

Name	Function	Source
Prokka 1.12	Rapid prokaryotic genome annotation tool	[24]
Roary 1.0	Rapid large-scale prokaryote pan genome analysis	[23]
BLAST+	Local alignment search	[66]
PSORTb 3.0	Prediction of protein subcellular localization	[30]
HMMTOP 2.1	Prediction of transmembrane topology	[45]
DEG	Database of essential genes to check essentiality.	[33]
VFDB	Virulence factors database for virulence identification	[39]
MvirDB	Microbial virulence database for virulence identification	[40]
RefSeq (Human Genome Resources)	Human genome database for Homology search	[44]
ABCPred	B-Cell epitope prediction	[49]
Propred-I	Prediction of promiscuous major histocompatibility complex (MHC) Class-I binding sites.	[50]
Propred	Prediction of MHC Class-II binding regions in an antigen sequence.	[51]
Vaxijen v2.0	Antigenicity checking	[18]
UniProt-SwissProt	Manually annotated protein sequences database with information extracted from literature for homology search and functional annotation	[53]
COG	Functional annotation	[54]
CARD	antibiotic resistance analysis	[55]

**Table 2:** Comparison of PanRV with other available tools on the basis of selected features

Features	Vaxign	NERVE	Jenner-predict	VacSol	PanRV
Pangenome Estimation	×	×	×	×	✓
Protein Localization Prediction	✓	✓	✓	✓	✓
Essential Genes Identification	**×**	**×**	**×**	✓	✓
Virulent Factor Identification	**×**	✓	✓	✓	✓
Homology Analysis with Human	✓	✓	✓	✓	✓
Homology Analysis with Gut Flora	✓	✓	✓	×	✓
Identification of Trans Membrane Helices	✓	✓	✓	✓	✓
Molecular Weight Estimation	**×**	**×**	**×**	×	✓
Epitope Mapping	✓	**×**	✓	✓	✓
Functional Annotation using COG	**×**	**×**	**×**	×	✓
Antibiotic Resistance Association Analysis	**×**	**×**	**×**	×	✓
Graphical User Interface	✓	**×**	✓	✓	✓
Downloadable Package	**×**	✓	**×**	✓	✓
Automatic Installer	**×**	**×**	**×**	✓	✓

× indicates absence while ✓ indicates the presence of a specific feature in the **respective tools**

### Implementation

**Fig. 1:**
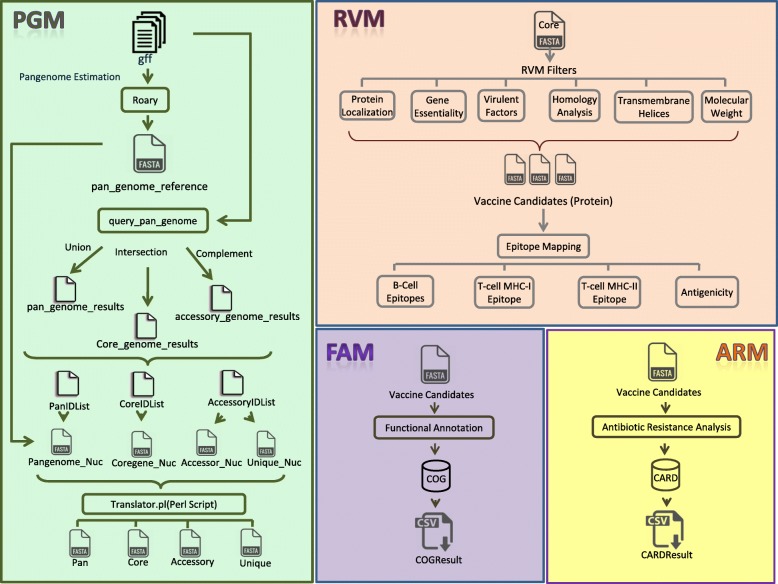
workflow of Pangenome-Reverse Vaccinology Package: Four modules of the pipeline (1) PGM (Pangenome Estimation Module), (2) RVM (Reverse Vaccinology Module), (3) FAM (Functional Annotation Module), (4) ARM (Antibiotic Resistance Association Module). PGM starts with multiple genomes files (.gff). These files are subjected to pangenome estimation pipeline (Roary), generating a pan_genome_reference along with several other supplementary files. Roary commands of query_pan_genome (union, intersection, complement) generate files (pan_genome_results, core_genome_results, accessory_genome_results). These files include gene ID (all isolates) in the respective category (Pan, Core, and Accessory) file. IDs lists (PanIDList, CoreIDList, AccessoryIDList) are picked from these files. IDs are then mapped to pan_genome_reference file and nucleotide FASTA sequences are extracted (Pangenome_Nuc, Coregene_Nuc, Accessor_Nuc, Unique_Nuc). Protein FASTA files (Pan, Core, Accessory, Unique) are generated by running a Perl script (Translator.pl). These predicted sets (Pan, Core, and Accessory) from PGM can be further subjected to RVM to identify putative vaccine candidates. Where selected pangenome category passes through each subfilter of RVM that extracts putative vaccine candidates along with their epitopes using the epitope mapping component. FAM and ARM identify functional annotation/significance and antibiotic resistance association of input FASTA file employing COG/UniProt and CARD databases, respectively. The results are displayed in CSV files

The functionalities of PanRV are elaborated as a workflow diagram in Fig. 1. Each module is further discussed below along with their specific functionality:

### Pangenome estimation module (PGM)

This module is designed to estimate the microbial pangenome including pan, core, dispensable and unique genes among multiple genomes. Roary [23] (rapid large-scale prokaryote pangenome analysis Pipeline) is integrated in PGM for pangenome estimation. Roary has the potential to generate the pangenome of thousands of prokaryotic strains in reduced time and less space complexity. Input for PGM is in gff format (files all isolates) generated through Prokka. Prokka is a rapid prokaryotic genome annotation tool. To avoid conflicting annotations from varied tools, it is suggested that annotation of all isolates (genomes) must be performed by Prokka 1.12 [24], prior to pangenome estimation.

The reason for the implementation of Roary is the utility as the existing pangenome estimation tools such as PGAP [25], PanOCT [26] and LS-BSR [27], the running time and memory usage increases exponentially with increasing dataset size, making large datasets computationally less feasible. Despite all the functional capabilities in Roary, yet a single default query in Roary is not enough to calculate genomic categories of pangenome (pan, core, dispensable and unique genes/proteins) in FASTA format protein sequences. Therefore, we developed an in house bash script that executes different steps of Roary sequentially and manipulates results to extract genomic categories (pan, core, dispensable and unique genes) in nucleotide FASTA format. Later, another script is executed to translate these nucleotide sequenced categories into protein sequences that can be further analyzed for vaccine targets identification using the RVM. Workflow of our PGM is elaborated in Fig. 1**.**

### Reverse vaccinology module (RVM)

The RVM can be executed sequentially along with the pangenome module or independently based on user interest. The input file subjected to this module is screened for potential vaccine candidates based on the RV parameters (discussed below). RVM incorporates sub-filters which are comprised of various efficient tools and updated databases to achieve optimal output. Each database and tool is downloaded and installed locally. BLAST searches are enabled for all the databases with defined threshold values provided in GUI. The default threshold values are set for each filter, however, the user may change these values to make strict or flexible through “select or de-select” individual filters according to their study requirement. Each sub-filter is further elaborated here.

#### Protein localization filter

Proteins found in the extracellular membrane, periplasmic membrane, and secreted proteins are selected by the filter. As these proteins involve in pathogen invasion and colonization into the host cell and play a major role in bacterial physiology and pathogenesis [28]. Secondly, these exoproteins are considered as an essential target of the adaptive immune response, therefore, these targets may be suggested as effective vaccine candidates [29]. Protein subcellular localization tool PSORTb 3.0. [30] has been employed in this filter to categorize the probable localization of the proteins. PSORTb is a broadly exploited and particular tool for predicting subcellular localization of proteins.

#### Gene essentiality filter

This filter explores and selects essential genes, which are indispensable to major cellular functions and viability of the organism. These genes have already been proved to be favorable drug targets in various pathogens [31–33]. Thus targeting these genes/proteins, may have a lethal effect on the microbe [34]. For this purpose Database of Essential Genes (DEG) [33] is employed as a filter. DEG is the first database of its kind to report essential genes and collects genes determined by genome-wide experiments.

#### Virulent factors filter

Virulent factors include proteins which are involved in pathogenesis and infection. Targeting these proteins as vaccine candidates will affect only pathogenic bacteria, thus increasing the vaccine efficiency [35–38]. Virulence Factor Database (VFdb) [39] and microbial virulence database (MvirDB) [40] have been integrated and used as a filter for the selection of virulent factors in the genome (proteomic data). VFdb is known to be an extensive warehouse of known bacterial virulence factors (VFs). It has delivered extensive and all-inclusive latest knowledge-based experimentally verified bacterial virulence factors.

#### Homology filter for human and gut flora

Homology filter selects only those proteins as vaccine candidates which are non-homologous to human and non-pathogenic bacteria from the gut flora (normal flora). This exclusion of homologs is required to avoid autoimmunity in the host [41] and to protect the symbiotic environment of gut flora [42]. Swiss-Prot [43] and RefSeq [44] BLAST searches are used for the identification of human homologs. Both the databases are unique in providing reliable annotation, consistent nomenclature, and direct links to specific databases with negligible redundancy. An internal database for 79 gut floral species [45] has been created to determine the possible homologies of the candidates with gut flora.

#### Trans-membrane helices filter

Transmembrane topology prediction server HMMTOP version 2.0 [46] has been incorporated to predict the number of transmembrane helices in a protein structure and selects the proteins having less than two transmembrane helices. As proteins with multiple transmembrane helices are difficult to purify otherwise, consequently not being considered efficient targets for vaccines [47]. The HMMTOP software operates on the basis of the hidden Markov model (HMM) and predicts transmembrane helices established on the difference in amino acid distributions in several structural portions of the proteins [46].

#### Molecular weight filter

This filter selects proteins having < 110 kDa molecular weight in the data set (proteome). As small (low mol. weight) proteins can easily be purified and handled effectively during vaccine development [45], a JAVA program based on the weight of amino acid sequences is incorporated into the module to compute molecular weights of candidates proteins. The program estimations are subject to cross-checking with various proteins from UniProt [48].

#### Epitope mapping filter

Proteins passed through all of the above parameters are considered as PVCs and are then subjected to this filter for the identification of immunogenic epitopes within these prioritized candidates. OSDDLinux (http://osddlinux.osdd.net) is used for antigenic epitope detection. It is a customized LINUX operating system which integrates open source software, libraries, workflows and web services in Linux for creating an environment for the drug discovery. OSDDLinux incorporated in this module provides multiple standalone programs like ABCPred [49], ProPred1 [50] and ProPred [51]. ABCPred is used to predict B cell epitope(s) by implementing artificial neural networks [52]. For the prediction of peptides that bind to MHC class-I alleles, ProPred1 is employed. ProPred predicts MHC class II binding regions in antigenic protein sequences (PVCs). Furthermore, the antigenicity of the selected epitopes is verified by Vaxijen v2.0 [18]. Epitopes that have values more than 0.4 (by default) are considered as potent antigenic.

### Functional annotation module (FAM)

Functional annotation is necessary as it reveals biological, cellular and molecular functional significance of the screened microbial targets. This information is critical for in-vivo testing development of candidate vaccines. For this purpose, functional annotation of candidate proteins is carried out through UniProt [53] and COG database 2014 [54] which are integrated into the FAM module. [7]. A protein FASTA file can be subjected to this module where the BLAST search is carried out against the COG database with user-defined threshold values provided in GUI. Both databases are employed due to their specific features; UniProt provides manually curated protein sequence information and functional detail [53] and the COG database is a famous tool used for performing functional annotation.

### Antibiotic resistance association module (ARM)

ARM efficiently detects the association of the predicted PVCs with antibiotic resistance. For this purpose, a comprehensive Antibiotic Resistance Database (CARD) [55] is incorporated into the pipeline. The CARD carries manually curated data and is considered as an advanced knowledge resource in the field of antibiotic resistance. Resistant determinants could be screen by BLAST search against CARD with threshold values in GUI. This module may also be used prior to RVM, where only those proteins identified to have antibiotic resistance association may only be subjected to RVM for anti-resistance vaccine candidates identification, the approach could also serve as an alternative to target the multi-drug resistant pathogens [56].

## Results

PanRV has proved to be the first comprehensive automated pipeline that can precisely and efficiently identify putative vaccine candidates from species pangenome. The pipeline is user-friendly as it has an interactive graphical interface and one step installation process through the designed installer. The pipeline is tested and validated by analyzing 301 genomes (strains) of *Streptococcus aureus (S. aureus)*. The pipeline with its all functional modules has been validated by experimentally known antigenic data. The complete input data is provided as **Input_Dataset.rar** in supplementary data while detailed results (files) are provided as **Results.rar** folder available at (https://sourceforge.net/projects/panrv2/).

The pangenome of 301 strains of *S. aureus* is estimated by PGM, which comprises of 11,384 pan, 1524 core, 6793 accessory, and 3067 unique genes families. The conserved core (1524 gene families) when subjected to RVM, 7 potential vaccine candidates (PVCs) are prioritized along with their immunogenic B-cell and T-cell epitopes. The list of identified candidate proteins their specific epitopes, functional significance (predicted through FAM) and any antibiotic resistance association (predicted through ARM) are shown in Table 3. The 5 out of 7 PVCs predicted are autolysin including three surface antigen ssaA2 (ssaA2_1, ssA2_2, ssA2_3), LysM domain repeat homologue of secretory antigens N-acetylmuramoyl-L-alanine amidase sle1 (sle_1), and LysM domain repeat homologue of Probable autolysin SsaALP, one Putative pyridoxine kinase, and one Serine protease Do-like HtrA. Experimental studies reveal that all of seven predicted PVCs are vital for bacterial cell survival, pathogenesis and exhibit immunogenicity in the host.

**Table 3:** List of vaccine targets prioritized via PanRV

RVM				ARM	FAM	
PanRV ID	Candidate Proteins (COG)	No. B cell Epitope	No. T cell Epitope	Resistance Association	COG ID	Function (UniProt)/annotation
95	Surface antigen	4	9	–	COG3942 M	ssaA2_1
169	LysM repeat	3	6	–	COG1388 M	N-acetylmuramoyl-L-alanine amidase sle1alanine amidase sle1
262	phosphomethylpyrimidine kinase	2	2	–	COG0351 H	Putative pyridoxine kinase
323	LysM repeat	5	6	–	COG1388 M	Probable autolysin SsaALP
998	Periplasmic serine protease, S1-C subfamily, contain C-terminal PDZ domain	2	2	–	COG0265 O	Serine protease Do-like HtrA
1303	Surface antigen	3	5	–	COG3942 M	Staphylococcal secretory antigen ssaA2_2
1306	Surface antigen	4	5	–	COG3942 M	Staphylococcal secretory antigen ssaA2_3

RVM (Reverse Vaccinology Module) results include PanRV IDs of 7 prioritized proteins along with the protein names and number of B and T cell epitopes. The results of ARM (Antibiotic Resistance Association Module) are illustrated as ARO IDs (if any) and the FAM (Functional Annotation Module) results are shown as COG IDs along with their functional annotations retrieved from UniProt

Surface antigens ssaA2 (PanRV IDs: 95,1303, 1306) are the core proteins of all available strains of *S. aureus* and associated with pathogenicity. Their immunogenicity has been proven by several experimental studies [57, 58]. Secretory antigen with LysM domain homologue of N-acetylmuramoyl-L-alanine amidase sle1 (PanRV ID: 169), is also predicted as a vaccine candidate. Sle1 belongs to a family of PGN hydrolases that localize to the septum during cell division where they exhibit peptidoglycan hydrolase activity, resulting in separation of the daughter cells [59, 60] subsequently increasing the No. of bacteria. Hence targeting Sle1 protein may prevent bacterial growth during infection. Mutagenesis studies reveal that deletion of *sle1* significantly reduces *S. aureus* extracellular vesicles (EVs) production. While microbial EVs influence the host-pathogen interaction during pathogenesis and are good immunogenic targets [61]. In a study [58] Sle1 and ssaA2 are recombinantly expressed, purified and tested for specific IgG responses using human plasma and study revealed high IgG response against *S. aureus* during infection. It implies that Sle1 and ssaA2 both are prime targets for the human immune system.

Hydroxymethylpyrimidine/phosphomethylpyrimidine kinase (PanRV ID: 262) is another PanRV identified candidate protein and is a homologue of *thiD*. It is involved in primary metabolism [58] in the thiamine biosynthetic process [59]. Thiamin (vitamin B1) is an important cofactor for all organisms in its active form thiamin diphosphate (ThDP) and *thiD* is an essential thiamin synthetic enzyme. It is also considered a promising drug target [60].

Another LysM domain repeat-containing protein is identified as PVC which is a homologue of Probable autolysin *SsaALP* (PanRV ID: 323). SsaALP is named for its similarity to the Staphylococcal secretory antigen A protein SsaA. It contained two repeating LysM domains, a motif also seen in other autolysins. A study examined its catalytic activity and proposed molecular engineering techniques to enhance its activity to act as a therapeutic target [58]. Likewise, serine protease Do-like HtrA (PanRV ID: 998) is predicted as PVC. HtrA proteins and their orthologues represent an important class of heat-shock-induced serine proteases and chaperones protecting protein structures which enhance bacterial survival under stress conditions [62] thus control the quality of proteins. It is the major virulence factor of bacteria that in many pathogenic bacteria strains lacking the HtrA function lose virulence or their virulence is decreased [63]. A whole genome approach study confirms this serine protease protein as a vaccine candidate against *S. aureus* [64].

All the identified candidate proteins exhibit notable biological significance as they contribute in major biological processes. Targeting these prioritized proteins might be detrimental to the survival of the bacteria. Thus, proteins prioritized as potential vaccine candidates through PanRV are evident as probable vaccine targets and are highly associated with bacterial survival and pathogenicity. Targeting these proteins could help in designing an effective and better broad-spectrum vaccine due to their conservation among all available isolates.

### Validation of PanRV

The results of PanRV are compared with other available tools and databases such as VacSol, Vaxign and Vaxgen for validation along with few experimental studies (Additional file 1). Core proteins (1524) identified by PanRV (through PGM) when subjected to VacSol, all of the PanRV predicted PVCs (7) are verified. When the same core protein analyzed through Vaxign a total of 19 PVCs are predicted. Upon comparative analysis, it has been revealed that only three of the PanRV identified PVCs (PanRV ID: 262, 1303, 1306) remain verified. The reason of remaining disagreements (16) is mainly due to the differences in PanRV and Vaxign filtering criteria, as Vaxign has not considered the candidate nature of being either essential or virulent (Additional file 1: Table S2). The predicted vaccine candidates (31) through Vaxgen database [65] (Vaccine-related Genes and Protective Antigens) when compared with PanRV predictions (Additional file 1: Table S3), results into a significant disagreements where 26 out of 31 antigens predicted are not the part of core (conserved protein set) proteome of the species, suggesting these antigens as not effective against all strains of *S. aureus*. Therefore, these antigens were disregarded by PanRV due to their narrow-spectrum. The remaining 5 proteins are also excluded by PanRV as they did not meet the criteria of being essential or virulence. If these filters (essential or virulence) in PanRV are turned off all the Vaxign predicted and experimentally verified antigens are selected as PVCs.

The overall validations of PanRV findings suggest that PanRV is more stringent towards conservation, virulence, and essentiality of antigenic proteins and therefore predict few candidates that are highly putative and can easily be processed for testing and experimental validation. Nevertheless, stringency parameters in PanRV can be customized by the user based on the study requirement. If users need to screen all antigens regardless of their importance in the survival of the pathogen and pathogenicity they may exclude essentiality and virulent factor determination filters, accordingly.

Results of the functional annotation module are verified through the NCBI online COG database (http://www.ncbi.nlm.nih.gov/Structure/bwrpsb/bwrpsb.cgi), and 100% of annotation was found similar. Both FAM and ARM are validated by respective databases COG and CARD, respectively.

### Performance of PanRV

**Fig. 2:**
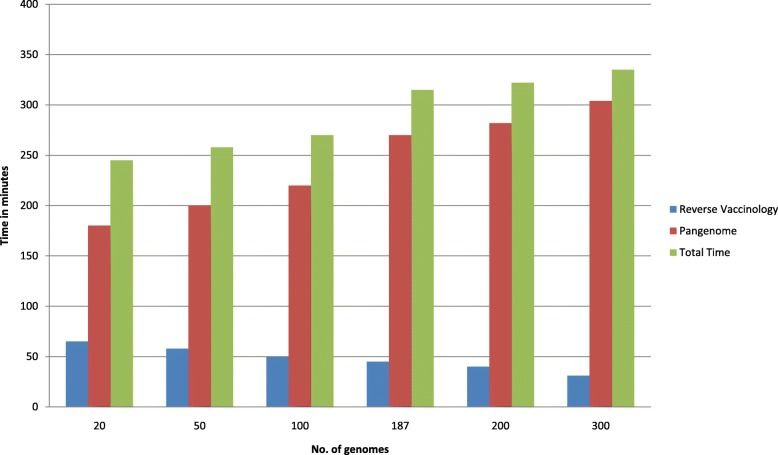
Comparison of the execution time of PanRV with a variable number of genomes of *Staphylococcus aureus*. PanRV execution time includes Pangenome analysis and Reverse vaccinology analysis. The graph depicts that with increase in number of genomes execution time steadily rises

Performance of PanRV is tested by the time is taken for pangenome and RV analyses of different No. of the genome. The pipeline is tested by using different variable (multiple) of genomes (100, 187, 200, and 301) maximum time taken for analysis on a 4 core system is 5 h 35 min. Time comparison versus a number of genomes shown in Fig. 2 depicts that with an increasing number of genome gradual increase in time of analysis is observed, nevertheless, the performance of the PanRV can be further enhanced by configuring it on a multi-node cluster thus making it feasible for big data analysis in reasonable time.

PanRV is a Linux based package developed in JAVA. The program works well on Ubuntu 14.04 and 16.04 with the latest JAVA version. However, PanRV has various dependencies and may require a 15GB of hard drive space. Similarly, for an uninterrupted analysis of large datasets, a 4GB RAM is recommended. PanRV provides an installer executable file (Installer.sh) to assist in installation (https://sourceforge.net/projects/panrv2/files/Installer.sh/download). By executing the installer, the required tools will be downloaded and installed accordingly. This feature is specially added for individuals with limited computational knowledge. The object-oriented programming is applied in this project and hence new features may also be added to improve and enhance the functionalities in the future. As the study of host-pathogen interactions and disease processes at the molecular level is considered significant for novel vaccine discovery process [19], therefore we intended to integrate host-pathogen interactions analysis in this pipeline to further enhance the specificity of predictions.

## Conclusion

PanRV is the first package implementing the pangenome and RV concepts together by integrating a number of standalone bioinformatics tools and databases. The PanRV is a user-friendly package with interactive analysis, predictions, and interpretations of results. It is currently a unique pipeline which provides utility to analyze multiple prokaryotic genomes (Pangenome), identifying the putative vaccine targets of broad-spectrum or species-specific nature. We expect that this pipeline will be useful to improve and accelerate the vaccine designing process against a broad range of pathogenic bacterial species. PanRV is currently available in a package form, and soon be launch as a web server to improve its accessibility and utility among the community.

## Availability and requirements

**Project name:** PanRV: Pangenome-Reverse Vaccinology package for identification of potential vaccine candidates.

**Project home page:** https://sourceforge.net/projects/panrv2/

**Archived version:** Not available.

**Operating system(s**): Linux.

**Programming language:** Java.

**Other requirements** (Pre Requisite Tools/Languages):

- NCBI BLAST+ [66]
- Prokka 1.12
- Roary 1.0
- PSORTb 3.0 [30]
- Hmmtop 2.1 [45]
- ABCPred [49]
- ProPred-I [50]
- ProPred [51]
- Java
- Perl
- Bioperl

**License**

Not applicable.

## Additional file

Additional file 1: Validation and comparison of PanRV Results. Additional file contains three tables. **Table S1** shows validation of seven putative vaccine candidates predicted by PanRV through experimental studies. **Table S2** shows comparison of PanRV with vaccine targets identified by Vaxign. **Table S3** includes experimentally known antigenic data from Vaxgen compared with PanRV. (DOCX 26 kb)

## Abbreviations

ARM: Antibiotic Resistance Analysis Module
BLAST: Basic Local Alignment Search Tool
COG: Cluster of Orthologues Groups
FAM: Functional Annotation Module
GUI: Graphical User Interface
MenB: *Neisseria meningitidis* serogroup B
PanRV: Pangenome Reverse Vaccinology Package
PGM: Pangenome Estimation Module
PVCs: Potential Vaccine Candidates
RV: Reverse Vaccinology
RVM: Reverse Vaccinology Module
*S.aureus*: *Staphylococcus aureus*
