# Supplementary material for: PanRV: Pangenome-reverse vaccinology approach for identifications of potential vaccine candidates in microbial pangenome
Source: BMC Bioinformatics. 2019 Mar 12;20:123. doi: 10.1186/s12859-019-2713-9 (PMC6419457; doi:10.1186/s12859-019-2713-9)
Supplement: Supplementary file 1 — Validation and comparison of PanRV Results. Additional file contains three tables. Table S1 shows validation of seven putative vaccine candidates predicted by PanRV through experimental studies. Table S2 shows comparison of PanRV with vaccine targets identified by Vaxign. Table S3 includes experimentally known antigenic data from Vaxgen compared with PanRV. (DOCX 26 kb) [file 12859_2019_2713_MOESM1_ESM.docx]

Validation and comparison of PanRV Results

**Additiona File 1**

**Table 1:** Validation of Seven PVCs predicted by PanRV

| **S.No** | **COG ID** |  | **COG Annotation** | **UniProt Annotation** | **References** |
| --- | --- | --- | --- | --- | --- |
| 95 | COG3942 | M | Surface antigen | ssaA2_1 | [[1](#_ENREF_1)] |
| 169 | COG1388 | M | LysM repeat | N-acetylmuramoyl-L-alanine amidase sle1alanine amidase sle1 | [[2-4](#_ENREF_2)] |
| 262 | COG0351 | H | Hydroxymethylpyrimidine/phosphomethylpyrimidine kinase | Putative pyridoxine kinase |  |
| 323 | COG1388 | M | LysM repeat | Probable autolysin SsaALP | [[1](#_ENREF_1)] |
| 998 | COG0265 | O | Periplasmic serine protease, S1-C subfamily, contain C-terminal PDZ domain | Serine protease Do-like HtrA | [[5](#_ENREF_5)] |
| 1303 | COG3942 | M | Surface antigen | Staphylococcal secretory antigen ssaA2_2 | [[4](#_ENREF_4)] |
| 1306 | COG3942 | M | Surface antigen | Staphylococcal secretory antigen ssaA2_3 | [[4](#_ENREF_4)] |

**Table 2:** Vaxign Identified PVCs cross checked with PanRV

| **#** | **Protein Accession** | **Localization** | **PanRV** |
| --- | --- | --- | --- |
| 1 | HLFGLHOD_00137_group_3062 | Extracellular | Non-Virulent |
| 2 | HLFGLHOD_00271_group_1659 | Outermembrane | Non-Ess  Non-Vir |
| 3 | HLFGLHOD_00297_hel | Outermembrane | Non-Vir |
| 4 | HLFGLHOD_00337_group_809 | Periplasmic | Non-Ess-Non-Vir |
| 5 | HLFGLHOD_00584_group_5585 | Extracellular | Non-Ess-Non-Vir |
| 6 | HLFGLHOD_00677_group_3865 | Extracellular | Non-Ess-Non-Vir |
| 7 | HLFGLHOD_00990_sspB | Extracellular | Non-Ess-Non-Vir |
| 8 | HLFGLHOD_00995_atl_1 | Extracellular | Non-Ess-  Molweight |
| 9 | HLFGLHOD_01034_group_3294 | Outer | Non-Ess-Non-Vir |
| 10 | HLFGLHOD_01473_group_10323 | Extra | Non-Vir |
| 11 | HLFGLHOD_01548_kipA_2 | Outer | Non-Ess-Non-Vir |
| 12 | HLFGLHOD_02042_sceD | Extracellular | Non-Vir |
| **13** | **HLFGLHOD_02240_ssaA2_2** | **Extracellular** | **Pass** |
| **14** | **HLFGLHOD_02244_ssaA2_3** | **Extracellular** | **Pass** |
| 15 | HLFGLHOD_02320_ydaG | Extra | Non-Ess-Non-Vir |
| 16 | HLFGLHOD_02407_dapF | Extra | Non-Ess-Non-Vir |
| 17 | HLFGLHOD_02482_ssaA2_4 | Extracellular | Non-Ess-Non-Vir |
| 18 | HLFGLHOD_02507_isaA | Extracellular | Non-Vir |
| **19** | **HLFGLHOD_00551_pdxK** | **Periplasmic** | **Pass** |

### Table 3: Vaxgen: Vaccine-related Genes and Protective Antigens cross checked with PanRV

|  | **Gene ID** | **Gene Name** | **Sequence Strain (Species/Organism)** | **Protein Name** | **Vaccines Involving this Gene** | **PanRV** |
| --- | --- | --- | --- | --- | --- | --- |
| 1 | [**878**](http://www.violinet.org/vaxgen/gene_detail.php?c_gene_id=878) | aroA | *Staphylococcus aureus* subsp. aureus ED98 | 3-phosphoshikimate 1-carboxyvinyltransferase | • [Staphylococcus aureus aroA mutant vaccine](http://www.violinet.org/vaxquery/vaccine_detail.php?c_vaccine_id=3149) | Non-Core |
| 2 | [**847**](http://www.violinet.org/vaxgen/gene_detail.php?c_gene_id=847) | ClfA | *Staphylococcus aureus* subsp. aureus str. Newman | clumping factor A | • [S. aureus ClfA Protein Vaccine](http://www.violinet.org/vaxquery/vaccine_detail.php?c_vaccine_id=1082)  • [S. aureus DNA vaccine pClfaSrtD13](http://www.violinet.org/vaxquery/vaccine_detail.php?c_vaccine_id=3895)  • [S. aureus DNA vaccine encoding Efb, FnbpA, ClfA, Cna](http://www.violinet.org/vaxquery/vaccine_detail.php?c_vaccine_id=3896) | Non-Core |
| 3 | [**846**](http://www.violinet.org/vaxgen/gene_detail.php?c_gene_id=846) | cna | *Staphylococcus aureus* | collagen adhesin | • [S. aureus CNA Protein Vaccine](http://www.violinet.org/vaxquery/vaccine_detail.php?c_vaccine_id=1083)  • [S. aureus DNA vaccine encoding Efb, FnbpA, ClfA, Cna](http://www.violinet.org/vaxquery/vaccine_detail.php?c_vaccine_id=3896) | Non Core |
| 4 | [**4180**](http://www.violinet.org/vaxgen/gene_detail.php?c_gene_id=4180) | Csa1A | *Staphylococcus aureus* | Csa1A |  | Non-Core |
| 5 | [**1732**](http://www.violinet.org/vaxgen/gene_detail.php?c_gene_id=1732) | efb | *Staphylococcus aureus* subsp. aureus VRS11b | extracellular fibrinogen-binding protein Efb | • [S. aureus DNA vaccine encoding Efb, FnbpA, ClfA, Cna](http://www.violinet.org/vaxquery/vaccine_detail.php?c_vaccine_id=3896) | HLFGLHOD_01096_fib_2 non Core |
| 6 | [**4168**](http://www.violinet.org/vaxgen/gene_detail.php?c_gene_id=4168) | Efb extracellular fibrinogen binding protein | *Staphylococcus aureus* | Efb extracellular fibrinogen binding protein |  | HLFGLHOD_01096_fib_2 non Core |
| 7 | [**4176**](http://www.violinet.org/vaxgen/gene_detail.php?c_gene_id=4176) | ess extracellular A (EsxA) | *Staphylococcus aureus* | ess extracellular A (EsxA) |  | 88 non-Ess |
| 8 | [**4177**](http://www.violinet.org/vaxgen/gene_detail.php?c_gene_id=4177) | ess extracellular B (EsxB) | *Staphylococcus aureus* | ess extracellular B (EsxB) |  | HLFGLHOD_00280_group_5545 non Core |
| 9 | [**4179**](http://www.violinet.org/vaxgen/gene_detail.php?c_gene_id=4179) | Fhud2 | *Staphylococcus aureus* | Fhud2 |  | 1169 HLFGLHOD_02226_fhuD_2 (Cytoplasmic Membrane)  Cello2go Periplamis  PanRV Passed except localization |
| 10 | [**4183**](http://www.violinet.org/vaxgen/gene_detail.php?c_gene_id=4183) | fibronectin-binding protein B FnBPB | *Staphylococcus aureus* | fibronectin-binding protein B FnBPB |  | HLFGLHOD_02439_group_38 (Non Core) |
| 11 | [**849**](http://www.violinet.org/vaxgen/gene_detail.php?c_gene_id=849) | FnbA | *Staphylococcus aureus* subsp. aureus NCTC 8325 | Fibronectin-binding protein A | • [S. aureus FnbA Protein Vaccine](http://www.violinet.org/vaxquery/vaccine_detail.php?c_vaccine_id=1084)  • [S. aureus DNA vaccine encoding Efb, FnbpA, ClfA, Cna](http://www.violinet.org/vaxquery/vaccine_detail.php?c_vaccine_id=3896) | HLFGLHOD_02440 fnbA_1  Non-Core |
| 12 | [**4173**](http://www.violinet.org/vaxgen/gene_detail.php?c_gene_id=4173) | FnbpA | *Staphylococcus aureus* | FnbpA |  | HLFGLHOD_02440 fnbA_1  Non-Core |
| 13 | [**4181**](http://www.violinet.org/vaxgen/gene_detail.php?c_gene_id=4181) | GapC | *Staphylococcus aureus* | GapC |  | HLFGLHOD_00741_gapA1 (Cytoplasmic ,human gut flora homologue) |
| 14 | [**4189**](http://www.violinet.org/vaxgen/gene_detail.php?c_gene_id=4189) | GST | *Staphylococcus aureus* | GST |  | Non-Core |
| 15 | [**4178**](http://www.violinet.org/vaxgen/gene_detail.php?c_gene_id=4178) | hla | *Staphylococcus aureus* | hla |  | KOEIBHHN_02402_group_2838 (Non-Core) |
| 16 | [**4171**](http://www.violinet.org/vaxgen/gene_detail.php?c_gene_id=4171) | HlgB | *Staphylococcus aureus* | HlgB |  | 1251 HLFGLHOD_02360_hlgB(Non-Essential) |
| 17 | [**893**](http://www.violinet.org/vaxgen/gene_detail.php?c_gene_id=893) | isdB | *Staphylococcus aureus* subsp. aureus str. Newman | iron-regulated heme-iron binding protein IsdB |  | HLFGLHOD_01069_isdB(Non-Core) |
| 18 | [**4170**](http://www.violinet.org/vaxgen/gene_detail.php?c_gene_id=4170) | IsdB | *Staphylococcus aureus* | IsdB |  | HLFGLHOD_01069_isdB(Non-Core) |
| 19 | [**4188**](http://www.violinet.org/vaxgen/gene_detail.php?c_gene_id=4188) | IsdH | *Staphylococcus aureus* subsp. aureus 71193 | IsdH |  | HLFGLHOD_01664_isdH (Non-Core) |
| 20 | [**4187**](http://www.violinet.org/vaxgen/gene_detail.php?c_gene_id=4187) | leukotoxin LukF | *Staphylococcus aureus* | leukotoxin LukF |  | LJMPPIKL_02313_lukF (Non-Core) |
| 21 | [**4186**](http://www.violinet.org/vaxgen/gene_detail.php?c_gene_id=4186) | LukS | *Staphylococcus aureus* | LukS |  | DOEELINF_01463_group_5184 (Non-Core) |
| 22 | [**848**](http://www.violinet.org/vaxgen/gene_detail.php?c_gene_id=848) | MecA | *Staphylococcus aureus* subsp. aureus MRSA252 | penicillin-binding protein 2 prime (PBP2a) | • [S. aureus DNA Vaccine encoding PBP2a Protein](http://www.violinet.org/vaxquery/vaccine_detail.php?c_vaccine_id=1085) | HLFGLHOD_00038_pbp (Non-Core) |
| 23 | [**4169**](http://www.violinet.org/vaxgen/gene_detail.php?c_gene_id=4169) | MntC | *Staphylococcus aureus* | MntC |  | 267 HLFGLHOD_00601_mntA  PanRV(CytoplasmicMembrane, Non-esse)  Cello2go periplasmic |
| 24 | [**4167**](http://www.violinet.org/vaxgen/gene_detail.php?c_gene_id=4167) | SasA | *Staphylococcus aureus* | SasA |  | EOLEMGPD_02931_group_121 (Non-Core) |
| 25 | [**4184**](http://www.violinet.org/vaxgen/gene_detail.php?c_gene_id=4184) | SdrD | *Staphylococcus aureus* | SdrD |  | HLFGLHOD_00534_sdrD (Non-Core) |
| 26 | [**4191**](http://www.violinet.org/vaxgen/gene_detail.php?c_gene_id=4191) | SEA | *Staphylococcus aureus* subsp. aureus SK1585 | SEA |  | JBAEKOOB_01504_group_253 (Non-Core) |
| 27 | [**4185**](http://www.violinet.org/vaxgen/gene_detail.php?c_gene_id=4185) | SEB | *Staphylococcus aureus* subsp. aureus COL | SEB |  | GJALMALH_00855_group_6181 (Non-Core) |
| 28 | [**4174**](http://www.violinet.org/vaxgen/gene_detail.php?c_gene_id=4174) | SpA | *Staphylococcus aureus* | SpA |  | GCMBAADG_02724_spa_1 (Non-Core) |
| 29 | [**4172**](http://www.violinet.org/vaxgen/gene_detail.php?c_gene_id=4172) | staphylococcal enterotoxin A | *Staphylococcus aureus* | staphylococcal enterotoxin A |  | GJALMALH_02074_entA_5 (Non-Core) |
| 30 | [**4182**](http://www.violinet.org/vaxgen/gene_detail.php?c_gene_id=4182) | target of RNAIII-activating protein TRAP | *Staphylococcus aureus* | target of RNAIII-activating protein TRAP |  | GBJDLPCM_01769_group_4301 (Non-Core) |
| 31 | [**4175**](http://www.violinet.org/vaxgen/gene_detail.php?c_gene_id=4175) | TSST-1 | *Staphylococcus aureus* | TSST-1 |  | [JBAEKOOB_02020_tst_3](https://blast.ncbi.nlm.nih.gov/Blast.cgi#alnHdr_Query_50116)(Non-Core) |

1. Etz, H., et al., *Identification of in vivo expressed vaccine candidate antigens from Staphylococcus aureus.* Proceedings of the National Academy of Sciences, 2002. **99**(10): p. 6573-6578.
2. Wang, X., et al., *Release of Staphylococcus aureus extracellular vesicles and their application as a vaccine platform.* Nature communications, 2018. **9**(1): p. 1379.

3. Szweda, P., et al., *Peptidoglycan hydrolases-potential weapons against Staphylococcus aureus.* Applied microbiology and biotechnology, 2012. **96**(5): p. 1157-1174.

4. Pastrana, F.R., et al., *Human antibody responses against non-covalently cell wall-bound Staphylococcus aureus proteins.* Scientific reports, 2018. **8**(1): p. 3234.

5. Weichhart, T., et al., *Functional selection of vaccine candidate peptides from Staphylococcus aureus whole-genome expression libraries in vitro.* Infection and immunity, 2003. **71**(8): p. 4633-4641.
